# Supplementary material for: Health system use among patients with mental health conditions in a community based sample in Toronto, Canada: A retrospective cohort study
Source: PLoS One. 2022 May 10;17(5):e0266377. doi: 10.1371/journal.pone.0266377 (PMC9089864; doi:10.1371/journal.pone.0266377)
Supplement: S1 Appendix — (DOCX) [file pone.0266377.s001.docx]

S1 Appendix: Search criteria for mental health conditions in health databank collaborative

| **Mental health condition** | **Search criteria** |
| --- | --- |
| **Addiction onset between 2012-01-01 and 2019-04-01** | Encounter Billing diagnosis in (303,304) or Health Condition Free Text match [ADDICTION, USE DISORDER, DRUG DEPENDENCE] |
|  |  |
|  | ED Visit with diagnosis in (F10, F11, F12, F13, F14, F15, F16, F17, F18, F19) |
|  |  |
| **Anxiety onset between 2012-01-01 and 2019-04-01** | Encounter Billing diagnosis in (300) or Health Condition Free Text match [ANXIETY, PHOBIA, OCD, GAD, PANIC DISORDER, OBSESSIVE COMPULSIVE DISORDER] |
|  |  |
|  | ED Visit with diagnosis in (F40, F41, F42, F43) |
|  |  |
| **Bipolar onset between 2012-01-01 and 2019-04-01** | Encounter Billing diagnosis in (296) or Encounter Billing service code (Q020) or Health Condition Free Text match [BIPOLAR] |
|  |  |
|  | ED Visit with diagnosis in (F30, F31) |
|  |  |
| **Depression onset between 2012-01-01 and 2019-04-01** | Encounter Billing diagnosis in (309,311) or validated CPCSSN depression condition |
|  |  |
|  | ED Visit with diagnosis in (F32, F33, F34, F38, F39) |
|  |  |
| **Eating Disorders onset between 2012-01-01 and 2019-04-01** | Encounter Billing diagnosis in (307) or Health Condition Free Text match [ANOREXIA, BULIMIA, EATING DISORDER, EDNOS, ED-NOS] |
|  |  |
|  | ED Visit with diagnosis in (F50) |
|  |  |
| **Schizophrenia onset between 2012-01-01 and 2019-04-01** | Encounter Billing diagnosis in (295, 298) or Encounter Billing service code (Q021) or Health Condition Free Text match [SCHIZOPHREN,PSYCHOS] |
|  |  |
|  | ED Visit with diagnosis in (F20, F25, F29) |
|  |  |

ED: Emergency Department

Table S2: Adjusted rate ratios for health care utilization in hospital and primary care

| **Outcome** | **Patient characteristics** | **Rate ratio** | **95% CI (L,U)** | | **P-value** |
| --- | --- | --- | --- | --- | --- |
| Hospital visit | Age: 18-30 years vs. 81+ years | 1.06 | 1.00 | 1.13 | 0.0597 |
| Hospital visit | Age: 31-40 years vs. 81+ years | 0.73 | 0.69 | 0.77 | <.0001 |
| Hospital visit | Age: 41-50 years vs. 81+ years | 0.61 | 0.57 | 0.64 | <.0001 |
| Hospital visit | Age: 51-60 years vs. 81+ years | 0.50 | 0.47 | 0.53 | <.0001 |
| Hospital visit | Age: 61-70 years vs. 81+ years | 0.52 | 0.49 | 0.54 | <.0001 |
| Hospital visit | Age: 71-80 years vs. 81+ years | 0.63 | 0.59 | 0.66 | <.0001 |
| Hospital visit | Gender: Female vs. Male | 1.01 | 0.98 | 1.04 | 0.3466 |
| Hospital visit | Deceased: Yes vs. No | 3.14 | 2.91 | 3.38 | <.0001 |
| Hospital visit | Region: Urban vs. Rural | 1.79 | 1.54 | 2.08 | <.0001 |
| Hospital visit | Income Quintiles: 2 vs. 1 | 0.92 | 0.88 | 0.96 | 0.0001 |
| Hospital visit | Income Quintiles: 3 vs. 1 | 0.85 | 0.81 | 0.89 | <.0001 |
| Hospital visit | Income Quintiles: 4 vs. 1 | 0.69 | 0.66 | 0.73 | <.0001 |
| Hospital visit | Income Quintiles: 5 vs. 1 | 0.68 | 0.66 | 0.71 | <.0001 |
| Hospital visit | Addiction: Yes vs. No | 1.74 | 1.59 | 1.91 | <.0001 |
| Hospital visit | Anxiety: Yes vs. No | 1.28 | 1.23 | 1.33 | <.0001 |
| Hospital visit | Bipolar: Yes vs. No | 1.06 | 0.95 | 1.17 | 0.2927 |
| Hospital visit | Depression: Yes vs. No | 1.06 | 1.02 | 1.10 | 0.0057 |
| Hospital visit | Eating Disorder: Yes vs. No | 1.08 | 1.01 | 1.15 | 0.0216 |
| Hospital visit | Psychosis: Yes vs. No | 1.18 | 0.98 | 1.41 | 0.0729 |
| Hospital visit | No. of co-morbidities: 1 vs. 0 | 1.08 | 1.04 | 1.12 | 0.0001 |
| Hospital visit | No. of co-morbidities: 2 vs. 0 | 1.29 | 1.23 | 1.35 | <.0001 |
| Hospital visit | No. of co-morbidities: 3+ vs. 0 | 1.59 | 1.50 | 1.69 | <.0001 |
| Primary care visit | Age: 18-30 years vs. 81+ years | 1.25 | 1.19 | 1.33 | <.0001 |
| Primary care visit | Age: 31-40 years vs. 81+ years | 0.82 | 0.78 | 0.86 | <.0001 |
| Primary care visit | Age: 41-50 years vs. 81+ years | 0.78 | 0.74 | 0.82 | <.0001 |
| Primary care visit | Age: 51-60 years vs. 81+ years | 0.88 | 0.84 | 0.92 | <.0001 |
| Primary care visit | Age: 61-70 years vs. 81+ years | 0.96 | 0.92 | 1.00 | 0.0408 |
| Primary care visit | Age: 71-80 years vs. 81+ years | 1.06 | 1.01 | 1.10 | 0.0103 |
| Primary care visit | Gender: Female vs. Male | 1.19 | 1.16 | 1.22 | <.0001 |
| Primary care visit | Deceased: Yes vs. No | 1.06 | 0.98 | 1.13 | 0.1348 |
| Primary care visit | Region: Urban vs. Rural | 1.22 | 1.09 | 1.35 | 0.0003 |
| Primary care visit | Income Quintiles: 2 vs. 1 | 1.03 | 0.99 | 1.07 | 0.1053 |
| Primary care visit | Income Quintiles: 3 vs. 1 | 1.04 | 1.00 | 1.08 | 0.0311 |
| Primary care visit | Income Quintiles: 4 vs. 1 | 1.07 | 1.02 | 1.11 | 0.0016 |
| Primary care visit | Income Quintiles: 5 vs. 1 | 1.08 | 1.04 | 1.11 | <.0001 |
| Primary care visit | Addiction: Yes vs. No | 1.05 | 0.96 | 1.14 | 0.2775 |
| Primary care visit | Anxiety: Yes vs. No | 1.65 | 1.60 | 1.70 | <.0001 |
| Primary care visit | Bipolar: Yes vs. No | 0.90 | 0.82 | 0.98 | 0.0157 |
| Primary care visit | Depression: Yes vs. No | 1.17 | 1.13 | 1.21 | <.0001 |
| Primary care visit | Eating Disorder: Yes vs. No | 1.48 | 1.40 | 1.56 | <.0001 |
| Primary care visit | Psychosis: Yes vs. No | 1.18 | 1.01 | 1.38 | 0.0426 |
| Primary care visit | No. of co-morbidities: 1 vs. 0 | 1.48 | 1.43 | 1.52 | <.0001 |
| Primary care visit | No. of co-morbidities: 2 vs. 0 | 1.96 | 1.89 | 2.04 | <.0001 |
| Primary care visit | No. of co-morbidities: 3+ vs. 0 | 2.38 | 2.27 | 2.50 | <.0001 |
| Primary care visit or hospital visit | Age: 18-30 years vs. 81+ years | 1.44 | 1.37 | 1.51 | <.0001 |
| Primary care visit or hospital visit | Age: 31-40 years vs. 81+ years | 0.85 | 0.81 | 0.89 | <.0001 |
| Primary care visit or hospital visit | Age: 41-50 years vs. 81+ years | 0.81 | 0.78 | 0.84 | <.0001 |
| Primary care visit or hospital visit | Age: 51-60 years vs. 81+ years | 0.84 | 0.81 | 0.87 | <.0001 |
| Primary care visit or hospital visit | Age: 61-70 years vs. 81+ years | 0.92 | 0.89 | 0.96 | <.0001 |
| Primary care visit or hospital visit | Age: 71-80 years vs. 81+ years | 0.99 | 0.96 | 1.03 | 0.7755 |
| Primary care visit or hospital visit | Gender: Female vs. Male | 1.16 | 1.14 | 1.19 | <.0001 |
| Primary care visit or hospital visit | Deceased: Yes vs. No | 1.45 | 1.37 | 1.54 | <.0001 |
| Primary care visit or hospital visit | Region: Urban vs. Rural | 1.33 | 1.21 | 1.46 | <.0001 |
| Primary care visit or hospital visit | Income Quintiles: 2 vs. 1 | 1.01 | 0.97 | 1.04 | 0.7355 |
| Primary care visit or hospital visit | Income Quintiles: 3 vs. 1 | 1.01 | 0.97 | 1.04 | 0.6877 |
| Primary care visit or hospital visit | Income Quintiles: 4 vs. 1 | 0.99 | 0.95 | 1.02 | 0.4682 |
| Primary care visit or hospital visit | Income Quintiles: 5 vs. 1 | 0.99 | 0.96 | 1.02 | 0.3483 |
| Primary care visit or hospital visit | Addiction: Yes vs. No | 1.55 | 1.44 | 1.66 | <.0001 |
| Primary care visit or hospital visit | Anxiety: Yes vs. No | 1.66 | 1.61 | 1.70 | <.0001 |
| Primary care visit or hospital visit | Bipolar: Yes vs. No | 1.06 | 0.98 | 1.14 | 0.1570 |
| Primary care visit or hospital visit | Depression: Yes vs. No | 1.20 | 1.17 | 1.24 | <.0001 |
| Primary care visit or hospital visit | Eating Disorder: Yes vs. No | 1.43 | 1.37 | 1.50 | <.0001 |
| Primary care visit or hospital visit | Psychosis: Yes vs. No | 2.31 | 2.02 | 2.64 | <.0001 |
| Primary care visit or hospital visit | No. of co-morbidities: 1 vs. 0 | 1.45 | 1.41 | 1.49 | <.0001 |
| Primary care visit or hospital visit | No. of co-morbidities: 2 vs. 0 | 1.89 | 1.83 | 1.96 | <.0001 |
| Primary care visit or hospital visit | No. of co-morbidities: 3+ vs. 0 | 2.28 | 2.18 | 2.37 | <.0001 |

Table S3: Adjusted odds ratios for the diagnosis of mental health conditions

| **Patient characteristics** | **Odds ratio** | **95% CI (L,U)** | | **P-value** |
| --- | --- | --- | --- | --- |
| Age: 18-30 years vs. 81+ years | 1.87 | 1.68 | 2.08 | <.0001 |
| Age: 31-40 years vs. 81+ years | 1.15 | 1.04 | 1.27 | 0.0076 |
| Age: 41-50 years vs. 81+ years | 1.28 | 1.16 | 1.41 | <.0001 |
| Age: 51-60 years vs. 81+ years | 1.45 | 1.31 | 1.59 | <.0001 |
| Age: 61-70 years vs. 81+ years | 1.26 | 1.14 | 1.39 | <.0001 |
| Age: 71-80 years vs. 81+ years | 1.26 | 1.14 | 1.40 | <.0001 |
| Sex: Female vs. Male | 1.35 | 1.27 | 1.42 | <.0001 |
| Region: Urban vs. Rural | 1.14 | 0.89 | 1.48 | 0.2968 |
| Income Quintiles: 2 vs. 1 | 1.05 | 0.96 | 1.14 | 0.2985 |
| Income Quintiles: 3 vs. 1 | 1.05 | 0.96 | 1.15 | 0.2591 |
| Income Quintiles: 4 vs. 1 | 0.96 | 0.88 | 1.05 | 0.3885 |
| Income Quintiles: 5 vs. 1 | 0.97 | 0.90 | 1.05 | 0.4480 |
